# Automated Region of Interest-Based Data Augmentation for Fallen Person Detection in Off-Road Autonomous Agricultural Vehicles

**DOI:** 10.3390/s24072371

**Published:** 2024-04-08

**Authors:** Hwapyeong Baek, Seunghyun Yu, Seungwook Son, Jongwoong Seo, Yongwha Chung

**Affiliations:** 1 Department of Computer Convergence Software, Korea University, Sejong 30019, Republic of Korea; qorrns156@korea.ac.kr (H.B.); tidlsld44@korea.ac.kr (S.Y.); seojongwoong@korea.ac.kr (J.S.); 2 Info Valley Korea Co., Ltd., Anyang 14067, Republic of Korea; sso7199@invako.kr

**Keywords:** autonomous agricultural vehicles, fallen person detection, data augmentation, automated region of interest

## Abstract

Due to the global population increase and the recovery of agricultural demand after the COVID-19 pandemic, the importance of agricultural automation and autonomous agricultural vehicles is growing. Fallen person detection is critical to preventing fatal accidents during autonomous agricultural vehicle operations. However, there is a challenge due to the relatively limited dataset for fallen persons in off-road environments compared to on-road pedestrian datasets. To enhance the generalization performance of fallen person detection off-road using object detection technology, data augmentation is necessary. This paper proposes a data augmentation technique called Automated Region of Interest Copy-Paste (ARCP) to address the issue of data scarcity. The technique involves copying real fallen person objects obtained from public source datasets and then pasting the objects onto a background off-road dataset. Segmentation annotations for these objects are generated using YOLOv8x-seg and Grounded-Segment-Anything, respectively. The proposed algorithm is then applied to automatically produce augmented data based on the generated segmentation annotations. The technique encompasses segmentation annotation generation, Intersection over Union-based segment setting, and Region of Interest configuration. When the ARCP technique is applied, significant improvements in detection accuracy are observed for two state-of-the-art object detectors: anchor-based YOLOv7x and anchor-free YOLOv8x, showing an increase of 17.8% (from 77.8% to 95.6%) and 12.4% (from 83.8% to 96.2%), respectively. This suggests high applicability for addressing the challenges of limited datasets in off-road environments and is expected to have a significant impact on the advancement of object detection technology in the agricultural industry.

## 1. Introduction

The continuous increase in the global population and the rapid rise in demand for agricultural products are driving the need for higher productivity in the global agriculture industry. According to statistics from the International Food and Agriculture Organization, the world’s population is reported to reach around 9.7 billion by 2050 [1]. Simultaneously, with the recovery of demand for agricultural products after the COVID-19 pandemic, there has been a sharp increase in the demand for agricultural products [2], which indicates the necessity for increased agricultural production. However, challenges such as the lack of interest from the younger generation in developed countries, lower income compared to office jobs, labor shortages due to the difficulties in farming, and the increasing average age of the global agricultural population, as reported in worldwide agricultural statistics, are expected to have a negative impact on agricultural productivity [3]. Currently, agricultural vehicles are specialized for agricultural work challenges for older workers, making prolonged tasks difficult and requiring frequent physical movement to check the work status, which, in turn, increases the risk of collisions and overturn accidents with other workers or obstacles in the forward direction [4]. Although agriculture is a less recognized occupation, it is one of the most hazardous professions, and each year, numerous fatalities occur due to accidents related to agricultural vehicles [5]. To address these challenges, there is a growing need for autonomous agricultural vehicles [6]. Research in the object detection field is active for autonomous agricultural vehicles [7].

Autonomous agricultural vehicles enable automation, enhance productivity, and work quality, and contribute to cost savings through reduced labor input. Furthermore, it can perform tasks that are either impossible with present vehicles or dangerous for people, presenting possibilities for increased agricultural productivity and meeting the growing food demand of the rising world population. As the demand for autonomous agricultural vehicles increases, development is particularly focused on tractors, which are used in agriculture throughout the seasons. The Society of Automotive Engineers has defined and classified the autonomy levels of autonomous vehicles from Level 0 to Level 5, with Level 3 and above eliminating the driver’s responsibility for Object and Event Detection and Response during normal operation. In contrast to autonomous vehicles, the definition of autonomy levels for autonomous agricultural vehicles emphasizes tasks related to farming separately from driving and focuses on handling situations in off-road environments rather than on roads. To raise the autonomy level beyond Level 3, it is crucial to establish a surrounding environmental perception system for safety in autonomous agricultural vehicles.

This paper aims to contribute to an effective nearby environmental perception system by enhancing fallen-person detection monitoring using object detection technology. This is particularly beneficial in preventing fatal accidents caused by unexpected falls of elderly workers during work, due to illness, overconsumption, fatigue, or collisions with a vehicle. To effectively detect fallen persons in object detection technology, the issue of data scarcity needs to be addressed. One major problem arising from data scarcity is the degradation of generalization performance. Generalization performance refers to the model’s performance on data not observed during the learning process. Degradation of generalization performance leads to the problem of overfitting, where the model excessively adapts to the limited training dataset. Off-road environmental data for fallen people are very limited compared to on-road, and detection accuracy is low on test sets that were not observed during training. To overcome this, data augmentation techniques are needed to improve generalization performance [8]. Adequate data generated through data augmentation helps object detectors accurately detect objects in various situations.

**Figure 1:**
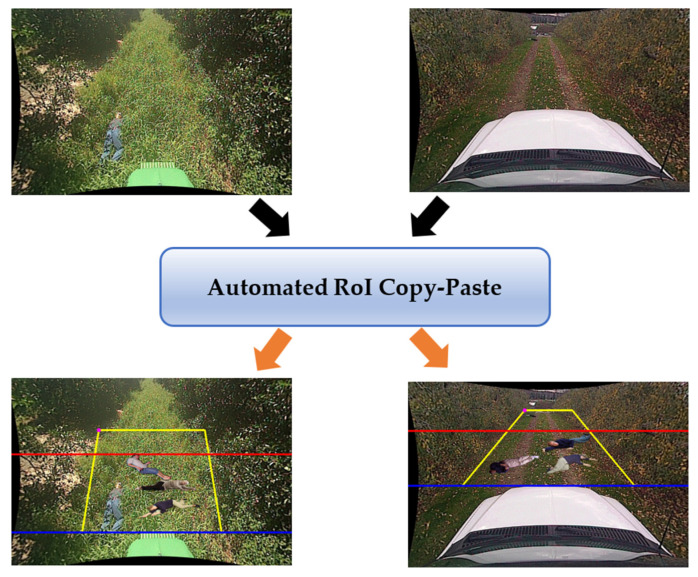
From background off-road images, augmented data for training is produced using the proposed ARCP. The black arrow indicates the input of the original images, and the orange arrow indicates the output of the augmented images to which this paper’s algorithm has been applied. Here the actual output image does not contain any dot and lines.

In this paper, we propose a new data augmentation technique called Automated Region of Interest Copy-Paste (ARCP), modifying the Copy-Paste [9]. Unlike conventional Copy-Paste, which randomly pastes objects from various source images into a background image for a specific object in the source image, ARCP automatically sets the Region of Interest (RoI) and pastes objects from multiple source images into the background image within that RoI. This technique works well in off-road environments without lane markings, as shown in Figure 1, and automatically sets RoIs in various environments. As shown in Figure 1, the yellow trapezoid represents the area symmetrically extended left and right after connecting the minimum and maximum bounding boxes within the interval. The purple point is the coordinate of the minimum bounding box. The red line is the minimum *y*-coordinate of the RoI set to reflect the tractor’s characteristic of having less need to detect people far away. The blue line is the *y*-coordinate of the bonnet. The area of the yellow trapezoid between the red and blue lines represents the RoI to be pasted, and its size is adjusted according to the ground truth.

Furthermore, this study utilizes the size and coordinates based on ground truth within segments of consecutive frames, enabling the utilization of any dataset based on segment-containing videos. For instance, crop datasets from agricultural environments and autonomous driving on-road datasets can be utilized. This paper specifically focuses on datasets concerning fallen person. Using Copy-Paste and instance segmentation models, you can automatically set realistic RoIs for any video-based data set, and it works in the real world.

The main contribution of this paper is to improve the accuracy of fallen person detection by Copy-Paste the scarce fallen person dataset in an off-road environment into the RoI through the proposed ARCP technique to build an ambient environment recognition system. There is no previous study that augments fallen-person data in an off-road environment. In addition, an existing study [10] that sets the RoI of a hazardous area based on the braking distance of a tractor in an off-road environment suffers from the inconvenience of requiring a manual RoI setting for each vehicle. In contrast, in this paper, we propose a technique to set the RoI automatically.

## 2. Related Work

### 2.1. Object Detection in Off-Road Environment

While there has been extensive research on object detection in on-road scenarios, and thus the corresponding public datasets, particularly in the context of autonomous driving applications [11,12,13,14,15,16,17,18,19,20,21,22,23,24,25], there is a noticeable scarcity of studies addressing object detection in off-road environments due to data limitations. For instance, a study by [26] evaluates a person detection algorithm in off-road environments, considering occlusion and non-standard poses. This study tests three image-only algorithms (Aggregate Channel Features, Deformable Parts Model, the Convolutional Neural Network) and discusses the sensitivity of performance metrics, particularly in high background texture and occlusion. Another study by [10] focuses on person hazard prevention, proposing new metrics for people detection in construction sites and off-road environments. It introduces safety-aware metrics combining practical variables related to person safety, an extension of the stixel algorithm, and a new detection robustness test based on a multi-object tracker. Meanwhile, ref. [27] explores computer vision architectures for real-time object detection in off-road environments, emphasizing multimodal deep fusion and sensor processing. It compares the SqueezeSeg architecture with a focus on data collection and semantic ground truth obtained using the Mississippi State University Autonomous Vehicle Simulator, demonstrating improvements in SqueezeSeg performance metrics. In contrast, ref. [28] introduces a domain-randomized synthetic image generator for training deep neural networks in the context of vehicle detection in off-road environments. This paper particularly focuses on off-road army tank detection.

There are no published studies addressing the improvement in detection accuracy for fallen persons in off-road environments. For fallen persons (i.e., object classes that are not often seen on-road as well as off-road), the synthetic quality of “generative” AI, such as Generative Adversarial Networks (GAN) [29] or the diffusion model [30] may not be satisfied. In this context, our approach proposes a data augmentation technique utilizing individual segmentation methods to enhance the detection accuracy for “real” fallen persons in off-road environments.

### 2.2. Data Augmentation for Object Detection

Data augmentation serves various purposes to enhance the generalization performance of image processing applications [31]. Particularly, modern object detectors apply not only basic augmentation techniques, such as random brightness, contrast, scaling, cropping, flipping, and rotation, but also advanced augmentation techniques, like MixUp [32], CutMix [33], and Mosaic [34]. Furthermore, there is a data augmentation technology called RandAug [35], which evolved from AutoAugment. This technique involves generating data by randomly selecting from a list of image transformations, including cropping, scaling, rotation, and color adjustments. Unlike the previous AutoAug, RandAug proposes a more competitive technique through parameterization without a separate data augmentation policy. On the other hand, research on synthetic images using GAN and the diffusion model has also been conducted [36,37,38,39,40,41,42,43]. Especially, the diffusion model, a probabilistic generative model that generates and restores noise during training to create images, is recently used for image generation because it is well known that the synthetic qualities of diffusion models are much better than those of GAN [44].

Recently, the data augmentation technique specific to individual segmentation, Copy-Paste, has been actively researched across various application domains due to its intuitive nature and high performance [45,46,47]. It involves augmenting data by pasting objects from one real image onto a different background image after various transformations (crop, resize, and rotate). This technique is utilized to enhance the generalization performance of learning models in situations where acquiring data is challenging. With the expected advancement of Copy-Paste, it is anticipated that the high patch-level realism with other datasets will contribute to improving the generalization performance of learning models [48].

### 2.3. Instance Segmentation

Instance segmentation is a computer vision task that involves identifying and classifying objects based on pixels within an image, including the process of detecting the boundaries of each object. The goal of instance segmentation is to generate pixel-level segmentation annotations for an image, where each pixel is assigned to a specific object instance. This approach effectively addresses the challenge of manually creating a significant number of segmentation annotations for datasets by automating the generation of instance-specific annotations. To create an instance segmentation model that generates high-accuracy segmentation annotations, pre-training on a large-scale dataset with high generalization performance is essential. Commonly used pre-trained models and datasets include COCO [49] and Segment Anything (SAM) [50].

Grounded-Segment-Anything (Grounded-SAM) [51] is a project that combines the strengths of Grounding DINO [52] and SAM to address complex problems with the goal of individual segmentation. This paper utilizes Grounded-SAM, combining the advantages of Grounding DINO in enabling object detection for new classes without segmentation annotations and the benefits of SAM in leveraging a large dataset, for instance, segmentation models. This combination is employed for data augmentation in object detection.

In contrast to other studies that require a manual element to set the RoI, this study differs by automatically setting the size and coordinates of the RoI and proposes to utilize the RoI automatically combined with traditional Copy-Paste using a modern instance segmentation model.

## 3. Materials and Methods

### 3.1. Framework

**Figure 2:**
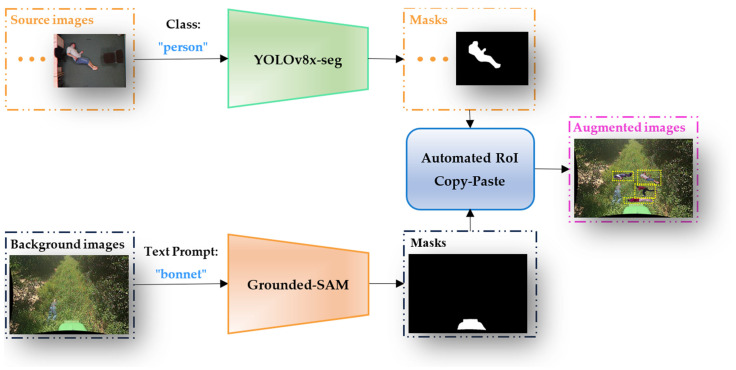
Framework of the ARCP pipeline. Objects with yellow dashed boxes in the augmented image represent objects pasted from the source images to the background image.

Figure 2 presents the overall framework of the new data augmentation proposed in this paper. The goal is to augment data for fallen person detection in off-road environments by utilizing segmentation annotations generated by an instance segmentation model. The newly proposed ARCP algorithm is then employed to create synthetic images based on these segmentation annotations. Multiple source images are used to generate segmentation annotations for standing or fallen persons using the YOLOv8x-seg [53] instance segmentation model pre-trained on the COCO dataset. Subsequently, the background off-road images are employed with Grounded-SAM to create segmentation annotations for the bonnet. Using these generated segmentation annotations, the proposed ARCP algorithm automatically generates RoIs. Finally, objects to be pasted within the generated RoIs are calculated to avoid overlapping, and the images are automatically augmented to create training off-road images that contribute to the learning process.

### 3.2. Automated RoI Copy-Paste (ARCP)

**Figure 3:**
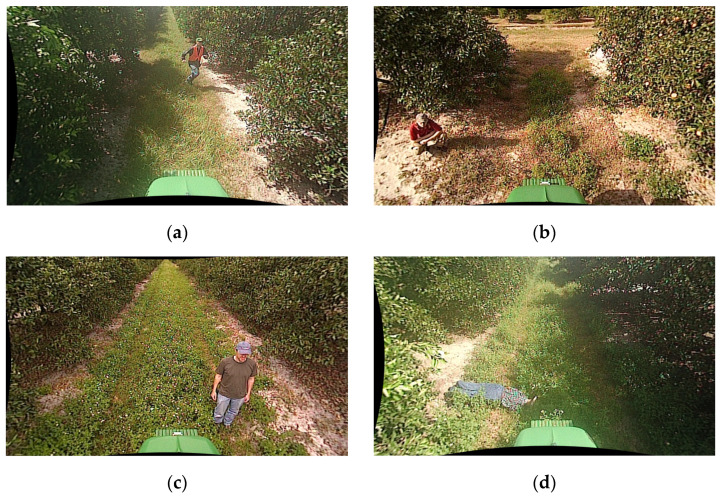
Various poses in scene composition for scene-by-scene in the NREC Person Detection Dataset [54]. (**a**) image containing an object in a running pose, (**b**) image containing an object in a sitting pose, (**c**) image containing an object in a standing pose, and (**d**) image containing an object in a fallen pose.

Figure 3 depicts a collection of videos featuring people in various poses such as jumping, sitting, standing, and fallen positions. These videos were captured at different locations and time frames, and as they are not from continuous time periods, the RoI changes with scene transitions. Therefore, it is necessary to identify the points where scenes transition to effectively handle the changing RoIs.

Assuming the t frame is the current video frame and the t + 1 frame is the next video frame, the ground truth annotations for both frames are utilized. If the Intersection over Union (IoU) value for the ground truth boxes of the two frames is greater than 0, indicating an overlap, the segments are considered to belong to the same scene, and the corresponding segment is set. Within each acquired segment, the RoI is set using information from the maximum and minimum bounding boxes of all boxes in that segment.

**Figure 4:**
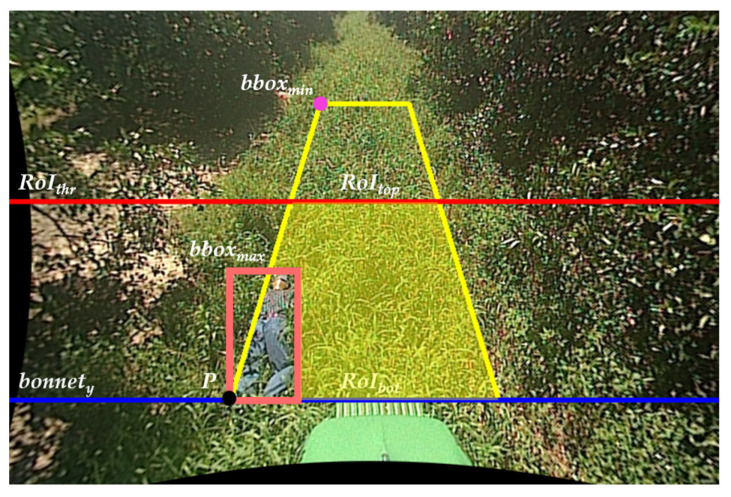
RoI setting of ARCP.

Subsequently, using Grounded-SAM, the mask for the bonnet is created, and the *y*-coordinate of this mask is determined. Using this information, the intersection point *P* between the maximum bounding box, *bbox_max_*, as shown in Figure 4, and the *y*-coordinate of the bonnet, *bonnet_y_*, is calculated. Next, a trapezoid is formed by symmetrically extending a line connecting the purple point, *bbox_min_*, and *P*. Then, using Equation (1), the *RoI_ratio_* is determined based on the ratio of the length of the base to the length of the top of this trapezoid. *I_w_* represents the width of the image, and *bbox_min_x_* and *bbox_max_x_* are the *x*-coordinates of *bbox_min_* and *bbox_max_*, respectively. The red line in Figure 4 represents the RoI threshold, determining the top of the RoI.

(1) $${RoI}_{ratio}=\left|\frac{{(I}_{w}-{bbox}_{min\_x})-\;{bbox}_{min\_x}}{{(I}_{w}-{bbox}_{max\_x})-\;{bbox}_{max\_x}}\right|$$

**Figure 5:**
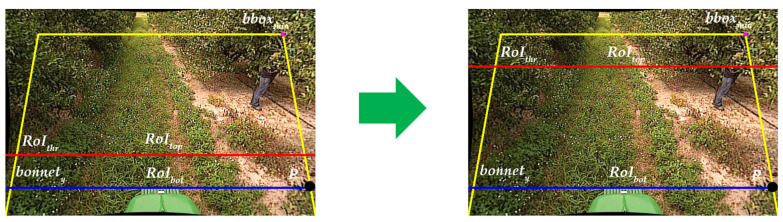
Complement the problem of Equation (1) using Equation (2).

However, as depicted in Figure 5, when the *bbox_min_x_* of a person appearing within the segment is closer to the left or right edge, the trapezoid becomes more rectangular. Consequently, the *RoI_ratio_* value increases. This results in a higher RoI threshold value, *RoI_thr_*, calculated in Equation (3), leading to a smaller range for the RoI. This issue arises even in scenarios where there is a potential risk of collision with the tractor, as the RoI cannot be properly set.

To address this issue, Equation (2) is employed. When the *RoI_ratio_* value exceeds 0.5, the operation (1 − *RoI_ratio_*) is performed. This means that as the *RoI_ratio_* increases, the reduction rate of the RoI area after the halfway point is lessened, effectively compensating for the problem described earlier.

(2) $${RoI}_{ratio}=1-{RoI}_{ratio},\;\;{RoI}_{ratio}>0.5$$

Next, Equation (3) is used to calculate the new y-coordinate, *RoI_thr_*, for the RoI. *RoI_thr_* represents the minimum *y*-coordinate for the RoI, focusing on the necessary area for data augmentation in a tractor environment where a short braking distance means there is less needed to detect objects far away. In this equation, bonnety corresponds to the *y*-coordinate of the bonnet, and *bbox_min_y_* is the *y*-coordinate of *bbox_min_*.

(3) $${RoI}_{thr}=\left|{bonnet}_{y}-b{box}_{min\_y}\right|\;*\;{RoI}_{ratio}+{bbox}_{min\_y}\;$$

*RoI_thr_* and the line where the trapezoid intersects with *RoI_thr_* are set on the top side of the RoI, denoted as *RoI_top_*. A trapezoid is then formed by connecting the previously obtained *RoI_top_* and *RoI_bot_*, excluding the areas occupied by the bonnet and the original person bounding box. The algorithm for segmenting each segment and setting the maximum and minimum bounding boxes is outlined in Algorithm 1.
**Algorithm 1.** Set Up Maximum and Minimum Bounding Boxes for Each Section**Input:**
   - ‘*bg_imgs*’: List of background images
   - ‘*bg_txts*’: List of text files corresponding to ‘*bg_imgs*’
**Output:**
   - ‘*results*’: Dictionary of containing bounding box statistics
**For each** ‘*bg_img*’ **in** ‘*bg_imgs*’ **do**   Open the corresponding file in ‘*bg_txts*’ and read lines into ‘*bg_lines*’    **For each** ‘*bg_line*’ **in** ‘*bg_lines*’ **do**     Parse ‘*x*’, ‘*y*’, ‘*w*’, ‘*h*’ as floats from ‘*bg_line*’     Adjust ‘*y*’ to (‘*y*’ − ‘*h*’/2)     Create ‘bbox’ as a tensor [‘*x*’, ‘*y*’, ‘*w*’, ‘*h*’]     Calculate ‘*area*’ as *‘w*’ * ‘*h*’     Increment ‘*count*’     Update bounding box statistics:       ‘*y_min_*’, ‘*y_max_*’, ‘*bbox_min_*’, ‘*bbox_max_*’ based on ‘*y*’       ‘*area_max_*’, ‘*width_max_*’, ‘*height_max_*’, ‘*width_min_*’, ‘*height_min_*’ based on ‘*w*’, ‘*h*’, and ‘*area*’     **If** ‘*prev_bbox*’ **exists, then**       Calculate ‘*iou*’       **If** ‘*iou*’ **equals 0, then**         Store current statistics in ‘results’ for ‘section_num’         Reset statistics         Increment ‘*section_num*’     Set ‘*prev_bbox*’ to ‘*bbox*’    Store final statistics in ‘results’ for the last ‘*section_num*’ **Return** ‘*results*’

Here is the proposed algorithm for pasting standing and fallen individuals using the maximum and minimum bounding box information obtained from Algorithm 1. Unlike conventional Copy-Paste, this algorithm pastes objects from multiple source images within the RoI using a specified maximum paste object value. This value is determined for each augmented image to ensure that it does not exceed the maximum limit and does not overlap. The algorithm is outlined in Algorithm 2.
**Algorithm 2.** Copy-Paste Objects from Multiple Images**Input:**
   - ‘*results*’: Output of Algorithm 1
   - ‘*fallen_person_imgs*’: List of fallen person images
**Output:**
   - Augmented image
**For each** ‘*bg_img*’ **in** ‘*bg_imgs*’ **do**   Initialize an ‘occlusion_mask’ to 0   *I_w_*, *I_h_* = size of ‘bg_img’   Open corresponding file in ‘*bg_txts*’, read lines into ‘*bg_lines*’    **For each** ‘*bg_line*’ **in** ‘*bg_lines*’ **do**      Update the corresponding ‘*occlusion_mask*’ to 255    **If the** section changes, **then**      Update ‘*height_max_*’, ‘*width_max_*’, ‘*height_min_*’, ‘*width_min_*’, ‘*area_max_*’      Create a ‘*bonnet*’ mask with Grounded-SAM   ${RoI}_{ratio}=\left|\frac{{(I}_{w}-{bbox}_{min\_x})-\;{bbox}_{min\_x}}{{(I}_{w}-{bbox}_{max\_x})-\;{bbox}_{max\_x}}\right|$
   **If** ‘${RoI}_{ratio}$’ > 0.5, **then**      ${RoI}_{ratio}=1-{RoI}_{ratio}$      ${RoI}_{thr}=\left|{bonnet}_{y}-b{box}_{min\_y}\right|\;*\;{RoI}_{ratio}+{bbox}_{min\_y}$
   **For each** ‘*fallen_person_img*’ **in** ‘*fallen_person_imgs*’ **do**      Break if you encounter the maximum paste object value during the loop       Create a ‘*fallen person*’ mask with the YOLOv8x-seg      Adjust position and size for fallen person within RoI      Randomly rotate ‘*fallen person*’ with a probability of 0.25      Each ‘*fallen person*’ is flipped vertically and horizontally with a probability of 0.5      **if** ‘*occlusion_mask*’ **exists** at the current position, **then**         **continue**      Paste a ‘*fallen person*’ into the background image      Apply alpha blending    Save the augmented image

Whenever the segment of the video changes, the bonnet mask image is generated using the transformer-based model, Grounded-SAM. For the standing or fallen person to be pasted within the RoI, we apply a rotation of 90, 180, or 270 degrees with a probability of 0.25, and a flip transformation is applied with a probability of 0.5 for both vertical and horizontal directions. This enhances generalization performance by accommodating various data transformations. During pasting, alpha blending is applied to improve the patch-level representation within the bounding box, aiming to enhance the overall visual quality [48].

## 4. Experimental Results and Discussion

### 4.1. Experimental Setup

In this paper, the NREC Person Detection Dataset [54], designed for detecting person objects in off-road environments, was utilized as the background image. The source datasets included the Fall Detection Dataset [55], the Fall Detection Dataset [56], and the UR Fall Detection Dataset [57], encompassing behavioral datasets with both fallen and standing person. The NREC Person Detection Dataset consists of images with a single person object, and the original image size is 720 × 480 pixels. For training and testing, a total of 28,479 images (1187 images with fallen persons) were selected for the training set, 449 images with fallen persons for the validation set, and 992 images for the test set. The standing and fallen person datasets from the Fall Detection Dataset, the Fall Detection Dataset, the UR Fall Detection Dataset were selected for training, consisting of 183, 2367, and 4576 images, respectively, totaling 7126 images. Additionally, to account for the characteristics of short braking distance tractors and to exclude small objects that may not be discernible to the person eye due to their distance from the camera, objects with small sizes were excluded. For a more accurate evaluation of the object detector, objects outside the RoI were also excluded for both the validation and test sets.

The object detectors used in the experiments were anchor-based YOLOv7x [58] and anchor-free YOLOv8x. Particularly, YOLOv7x and YOLOv8x apply not only basic augmentation techniques such as random brightness, contrast, scaling, cropping, flipping, and rotation but also advanced augmentation techniques like MixUp, CutMix, and Mosaic. In the experiment, the maximum paste object value, which denotes the value for pasting objects onto the background images without overlapping, was optimized by experiments, and a GeForce RTX 2080 Ti was used.

### 4.2. Evaluation Metrics

In the application of object detection technology, the ratio of the detected bounding box that matches the annotation bounding box is referred to as the IoU. If IoU is 50% or higher, it is categorized as True Positive (TP), and if it is less than 50%, it is categorized as False Positive (FP). Additionally, the case where the annotation bounding box is not detected is referred to as False Negative (FN). The evaluation metric provided by *Precision* is given by Equation (4), and the evaluation metric for *Recall* is given by Equation (5).

(4) $$Precision=\frac{TP}{TP+FP}$$

(5) $$Recall=\frac{TP}{TP+FN}$$

Average Precision (AP) is the area under the Precision-Recall curve, with the *x*-axis represents *Recall*, and the *y*-axis representing *Precision*. It is widely used as an evaluation metric in many computer vision applications to quantitatively assess performance, using both *Recall* and *Precision*. The *F*1 *Score* is an evaluation metric that assigns equal weight to *Precision* and *Recall*, providing a single numerical measure of accuracy. Its equation is provided in Equation (6).

(6) $$F1\;Score=2\;*\;\frac{Precision*Recall}{Pricision+Recall}$$

### 4.3. Experimental Analysis

**Figure 6:**
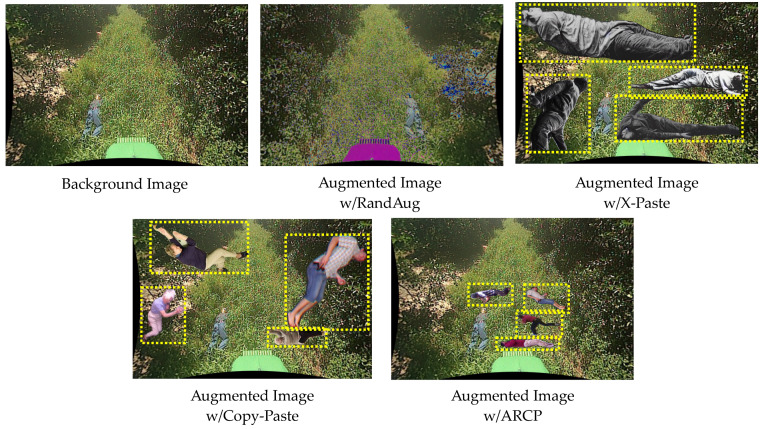
Illustration of Augmented Images from a Background Image (the NREC Person Detection Dataset [54]), with RandAug [35], X-Paste [42], Copy-Paste [9], and the proposed ARCP. Objects with yellow dashed boxes in the augmented image represent objects pasted from the source images to the background image.

Figure 6 represents augmented images of a fallen person object using the RandAug, X-Paste, Copy-Paste, and ARCP. The augmented image with RandAug, as mentioned below, is one of the images that has undergone several techniques using predefined parameters. This image has been modified with color adjustment and horizontal flipping. Due to the use of basic augmentation techniques with the same existing object, there is a limitation to the features that can be created from a dataset with a small number of objects. The quality of synthetic images created with X-Paste from augmented images with X-Paste is unsatisfactory. This issue arises from an insufficient dataset for the fallen person class during pre-learning, which increases the likelihood of learning incorrect features. Due to the limitations of the stable diffusion model in generating fallen person objects with limited training data, in the experiments, a total of 1296 synthetic images were used when pasting with X-Paste. Additionally, augmented images with Copy-Paste are randomly pasted at various coordinates and sizes, which raises the possibility of learning features of incorrect sizes due to the wrong background and placement within the box. In contrast, the proposed ARCP can learn features of the correct coordinates and size within the RoI.

Table 1 and Table 2 compare the results of models trained on datasets augmented using existing data augmentation techniques and the proposed ARCP. The accuracy of the YOLOv7x and YOLOv8x baselines was based on various data augmentation techniques such as MixUp, CutMix, and Mosaic. Therefore, applying RandAug further did not significantly improve accuracy. Additionally, a fallen person was not a commonly encountered object class, so even when a diffusion model was applied like X-Paste, it did not yield satisfactory quality in synthetic images, presenting a limitation. In contrast, Copy-Paste and ARCP, which generated images of fallen people off-road by extracting from real images of fallen people, measured relatively higher in accuracy.

According to the experimental results presented in Table 1, as a result of applying RandAug and X-Paste to YOLOv7x, both precision and recall were improved. Notably, RandAug exhibited a more pronounced increase in precision compared to recall. In contrast, while Copy-Paste and ARCP had lower increases in precision relative to recall compared to RandAug, both techniques showed a more substantial enhancement in precision. Significantly, ARCP achieved a considerable improvement with a precision of 97.3%. Additionally, its recall rate was also higher at 88.6%, surpassing other data augmentation methods in terms of overall accuracy. That is, when the ARCP was applied to the YOLOv7x model, the detection accuracy improved by 17.8%, from 77.8% to 95.6%, compared to the baseline. Additionally, when compared to the conventional Copy-Paste technique, the detection accuracy was improved by 6.9%, from 88.7% to 95.6%.

On the contrary, YOLOv8x applied more data augmentation techniques by default compared to YOLOv7x, resulting in a relatively higher measured accuracy for the baseline, and similar improvement effects were observed for YOLOv8x (see Table 2). Compared to the YOLOv8x baseline, RandAug slightly improved both precision and recall. On the other hand, X-Paste decreased precision but significantly increased recall. Copy-Paste significantly increased recall without decreasing precision, and ARCP significantly improved both precision and recall. That is, when applying the ARCP to YOLOv8x, the detection accuracy was improved by 12.4%, from 83.8% to 96.2%, compared to the baseline. Furthermore, when compared to conventional Copy-Paste, the detection accuracy was improved by 8.3%, from 87.9% to 96.2%.

Overall, YOLOv8 was designed with higher accuracy for detecting small objects compared to YOLOv7. It features an anchor-based architecture, multi-scaling prediction, and an improved backbone network. Consequently, in off-road environments where fallen people are often small and obscured by trees and grass, YOLOv8 demonstrated a higher baseline accuracy than YOLOv7. Furthermore, as evident from the results, it was observed that YOLOv7x exhibited a higher increase in accuracy compared to YOLOv8x when data augmentation was applied. This was evident as YOLOv8x reached over 70% baseline accuracy in just 50 epochs, whereas YOLOv7x required 100 epochs to achieve the same level of baseline accuracy above 70%. Additionally, the effectiveness of data augmentation techniques such as RandAug, X-Paste, Copy-Paste, and ARCP was reduced in YOLOv8x compared to YOLOv7x. Despite this, ARCP still delivered over 90% accuracy in terms of the previously described AP for both YOLOv7x and YOLOv8x.

**Figure 7:**
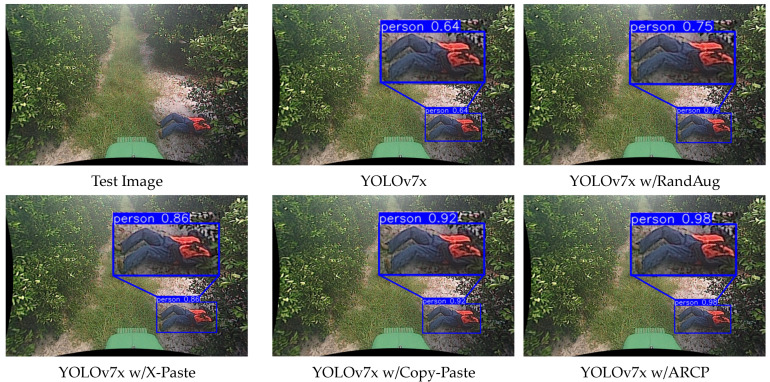
Detection results of YOLOv7x Baseline [58], RandAug [35], X-Paste [42], Copy-Paste [9], and ARCP.

**Figure 8:**
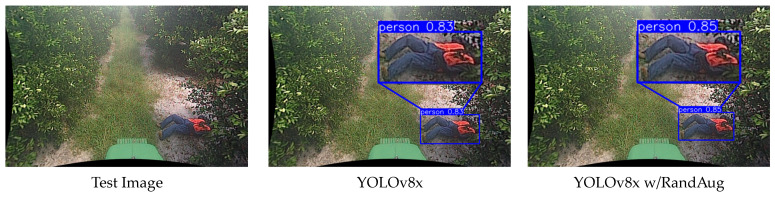

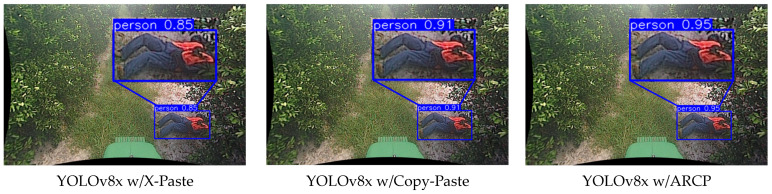
Detection results of YOLOv8x Baseline [53], RandAug [35], X-Paste [42], Copy-Paste [9], and ARCP.

For a comparative analysis between YOLOv7x and YOLOv8x, we compared the confidence scores of detection boxes for a test video detecting a fallen person using all techniques. The confidence score is a measure of how reliable the predicted bounding box is, expressed as a number between 0 and 1, with closer to 1 indicating higher confidence. In YOLOv7x, the baseline obtained a confidence score of 0.64, RandAug 0.75, X-Paste 0.86, Copy-Paste 0.92, and ARCP 0.98. Similarly, in YOLOv8x, the baseline obtained a score of 0.83, RandAug 0.85, X-Paste 0.85, Copy-Paste 0.91, and ARCP 0.95 (refer to examples in Figure 7 and Figure 8). In this paper, the proposed method, ARCP, achieved the highest confidence scores in both YOLOv7x and YOLOv8x, providing evidence of its superior effectiveness among various data augmentation techniques.

**Figure 9:**
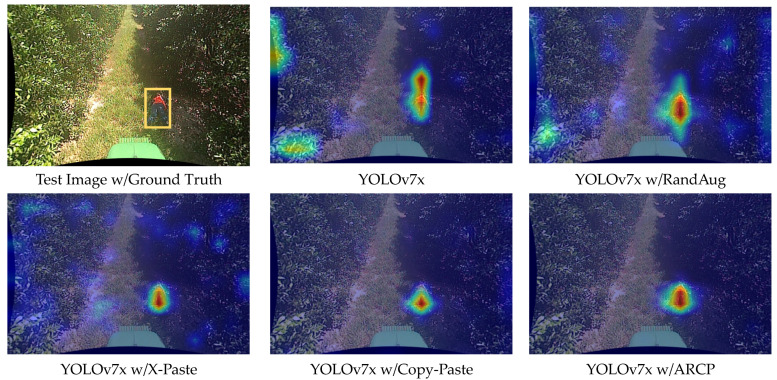
Heat map comparison of YOLOv7x baseline [58], RandAug [35], X-Paste [42], Copy-Paste [9], and ARCP with Grad-CAM. The yellow box represents the bounding box of the ground truth of the test image. The red areas indicate a stronger focus on the corresponding features when generating results, while the blue areas suggest a weaker emphasis.

**Figure 10:**
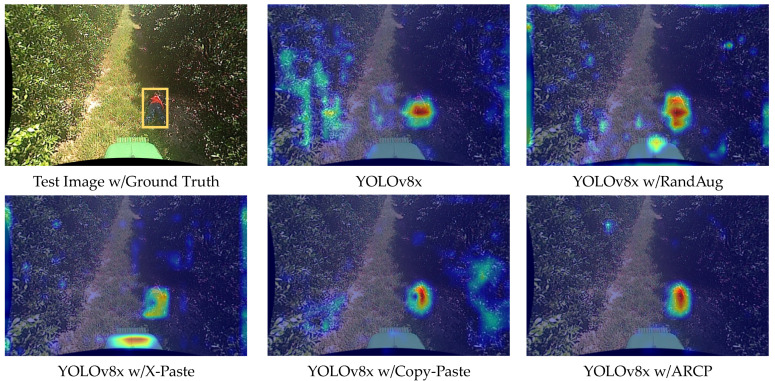
Heat map comparison of YOLOv8x baseline [53], RandAug [35], X-Paste [42], Copy-Paste [9], and ARCP with Grad-CAM. The yellow box represents the bounding box of the ground truth of the test image. The red areas indicate a stronger focus on the corresponding features when generating results, while the blue areas suggest a weaker emphasis.

Finally, to visualize the extent to which the models trained with each data augmentation technique focus on specific areas detecting fallen person, we compared images visualized using the Grad-CAM [59] technique for a fallen person test image (see example in Figure 9 and Figure 10). As shown in the figure, the red areas indicate a stronger focus on the corresponding features when generating results, while the blue areas suggest a weaker emphasis. As shown in the figure below, the red areas indicate that the model’s features learned in that area have a stronger emphasis when generating results, while the blue areas suggest a relatively weaker emphasis.

In the Grad-CAM shown in Figure 9, YOLOv7x demonstrated increases in both precision and recall across all data augmentations, using the lowest accuracy baseline, as seen in Table 1. RandAug, excluding the proposed ARCP, showed a significant improvement in precision as the width of the augmentation increased, displaying a spreading concentration around objects as seen in Grad-CAM. Conversely, X-Paste exhibited roughly double the increase in recall compared to precision, displaying the lowest accuracy among the compared data augmentation techniques. Additionally, Copy-Paste showed the second-highest accuracy in both YOLOv7x and YOLOv8x, with similar increases in precision and recall, significantly reducing the focus on false detections around objects. This affirmed that “real” objects were more beneficial than incorrectly generated objects due to data scarcity in the stable diffusion model’s pre-training. Modifying Copy-Paste to paste ARCPs into RoIs revealed a higher concentration within the RoI compared to other data augmentation techniques concentrating around trees and bonnets outside the RoI. It showed the highest performance in both precision and recall, with precision showing a greater increase than recall. Both YOLOv7x and YOLOv8x effectively centered their focus around objects, notably reducing false detections in surrounding areas. YOLOv8x tended to exhibit a wider distribution of yellow areas in Grad-CAM compared to YOLOv7x. Unlike YOLOv7x, RandAug showed a lower increase in precision compared to recall, and while X-Paste’s precision accuracy decreased significantly compared to the baseline, recall ranked second after ARCP. Features of incorrect object learning, as shown in Figure 10, led to bonnets being learned as human features, increasing false positives, and significantly decreasing precision. Copy-Paste slightly decreased from the baseline in precision but showed a greater increase in recall compared to precision. ARCP exhibited more focused concentration on objects compared to Copy-Paste. Furthermore, unlike YOLOv7x, YOLOv8x showed a larger increase in recall than precision with ARCP.

Among the various data augmentation techniques proposed in this paper, ARCP achieved the highest recall at 88.6% (YOLOv7x) and 91.0% (YOLOv8x). However, to reduce the incidence of the most critical safety incidents, such as fallen person, it is essential to explore methods that prioritize improving recall to the maximum extent possible, even if it results in a slight loss of precision. Thus, our method has contributed to demonstrating its effectiveness, particularly in detecting instances such as fallen person in low-data environments, notably in agriculture, rather than the stable diffusion model and conventional Copy-Paste techniques.

### 4.4. Discussion

Data augmentation is essential to improving the generalization performance of small datasets in object detection. This serves to mitigate the overfitting issue. To validate this effect quantitatively, it is imperative to verify the results across a test set comprising multiple unseen datasets. However, at present, there is no publicly accessible dataset apart from the NREC Person Detection Dataset, designed for evaluating object detection performance with the class of fallen person in off-road environments. Therefore, we opted to construct a test set that closely approximates real-world conditions by employing ARCP on an agricultural person detection dataset. The FieldSAFE [60] dataset utilized in the subsequent experiments underwent manual annotation, involving the labeling of 261 instances.

**Figure 11:**
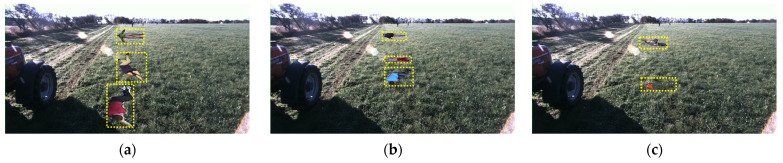
Images of test sets with ARCP applied using FieldSASFE [60] as a background image. The source images are (**a**) the Fall Detection Dataset [55] + Fall Detection Dataset [56], (**b**) the UR Fall Detection Dataset [57], and (**c**) the NREC Person Detection Dataset. Objects with yellow dashed boxes represent objects pasted from the source images to the background image.

Table 3 shows the results of the experiment to verify the resolution of the overfitting problem in the background. As shown in Figure 11, the FieldSAFE dataset was used as the test set by applying ARCP to the fallen person objects in the NREC Person Detection Dataset, which is the same as the previous test set, and the FieldSAFE dataset in the test set is 261 images, that is, 1414 instances.

From Table 3, we can see that the AP of baseline is 13.5%, which is relatively lower than the AP of Table 1, which clearly indicates that it is an unseen dataset. We observe that the Copy-Paste technique is 44.6%, which is 31.4 and 26% higher than RandAug and X-Paste, respectively, and ARCP, which improves the Copy-Paste technique, is 72.3%, which is 27.7% higher than Copy-Paste. This confirms that the proposed ARCP method effectively solves the overfitting problem for unseen environments.

In addition, Table 4 and Table 5 are experiments to verify the resolution of the object-specific overfitting problem during Copy-Paste. The alphabets in Table 4 and Table 5 refer to the datasets in Figure 11. The train set used for training is a dataset to which ARCP was applied using the NREC Person Detection Dataset as a background image and source images as multiple datasets, and the test set used for testing is a dataset to which ARCP was applied using the FieldSAFE dataset as a background image and source images as multiple datasets. This is the applied dataset. The Fall Detection Dataset has 183 images, so we combined it with the Fall Detection Dataset.

As demonstrated in Table 4, except for the accuracy on datasets (**a**) + (**b**) in YOLOv8x, it is found that ARCP is more effective in addressing overfitting for fallen person objects across multiple datasets in unseen environments compared to Copy-Paste.

**Figure 12:**
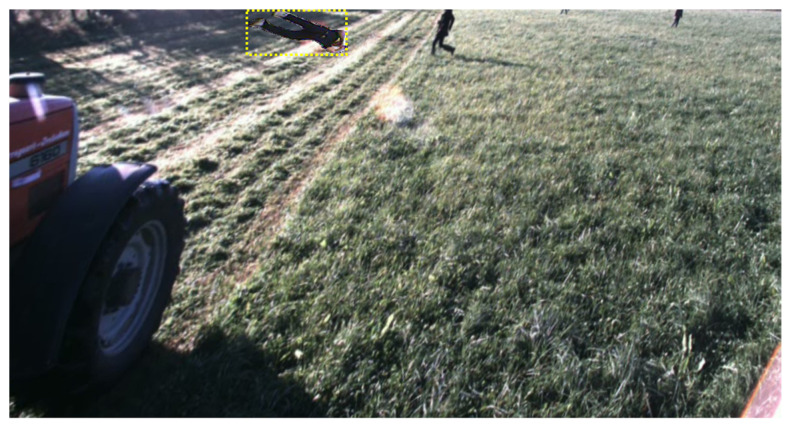
Image of the Test Set with ARCP applied to the FieldSAFE dataset with a warp perspective transformation in the background. Objects with yellow dashed boxes represent objects pasted from the source images to the background image.

Furthermore, when the proposed method involves Copy-Paste, it is crucial to consider the light intensity and angle adjustments to further alleviate the overfitting problem. As depicted in Figure 12, by applying a warp perspective transformation to the FieldSAFE dataset, we expressed light blurring and angle adjustments in the background. This was evaluated by using the fallen person dataset as the background image and the dataset with ARCP applied as the source image for testing. As shown in Table 6 and Table 7, it was confirmed that the ARCP technique achieved the highest accuracies of 34.2% and 19.0% in YOLOv7 and YOLOv8, respectively. This validates that ARCP performs better in environments with light blurring and distorted angles compared to Copy-Paste. This capability is particularly beneficial for detecting situations where the tractor’s body oscillates up and down during off-road driving.

Studying the consistency of light intensity and angle with real-world conditions during the process of Copy-Paste can enhance the similarity between the augmented dataset and the real dataset, thereby contributing to a more effective resolution of the data scarcity issue. This aspect warrants further investigation in future studies.

## 5. Conclusions

This paper aimed to enhance the environmental perception system of autonomous agricultural vehicles by improving the detection accuracy of fallen person through data augmentation. Specifically, to address the lack of datasets for fallen person in off-road environments, a new data augmentation technique called ARCP was proposed, which automatically augmented objects from multiple fallen person images onto a background off-road image, maximizing the RoI. ARCP utilized YOLOv8x-seg and Grounded-SAM for automatic segmentation masks, including IoU-based segment settings and RoI configuration.

Experimental results with fallen-person data in off-road environments showed that advanced augmentation techniques, such as MixUp, Cut-Mix, Mosaic, and RandAug (discovered through auto augmentation), had minimal impact. Diffusion-based synthetic data augmentation techniques also demonstrated less effectiveness than expected. However, both Copy-Paste and the proposed ARCP technique based on real data were found to be effective. In particular, the proposed ARCP technique showed significant improvements in detection accuracy, with an increase of 6.9% in YOLOv7x and 8.3% in YOLOv8x compared to Copy-Paste. This improvement in detection accuracy is expected to contribute to the leap in autonomous agricultural vehicles using object detection technology in the agricultural industry. The proposed technique is particularly anticipated to be highly applicable in datasets that are limited in off-road environments.

## Figures and Tables

**Table 1:** Accuracy in YOLOv7x [58] of data augmentation results among the RandAug [35], X-Paste [42], Copy-Paste [9], and ARCP.

Model	Precision↑ (%)	Recall↑ (%)	AP↑ (%)	F1 Score↑ (%)
YOLOv7x [58]	75.4	70.7	77.8	73.0
YOLOv7x w/RandAug [35]	93.0	76.2	87.0	83.8
YOLOv7x w/X-Paste [42]	79.0	79.1	84.0	79.0
YOLOv7x w/Copy-Paste [9]	84.9	82.9	88.7	83.9
YOLOv7x w/ARCP	**97.3**	**88.6**	**95.6**	**92.7**

* ↑ indicates that the higher the number, the better the performance. * Bold indicates the best number in this table.

**Table 2:** Accuracy in YOLOv8x [53] of data augmentation results among the RandAug [35], X-Paste [42], Copy-Paste [9], and ARCP.

Model	Precision↑ (%)	Recall↑ (%)	AP↑ (%)	F1 Score↑ (%)
YOLOv8x [53]	87.9	75.0	83.8	80.9
YOLOv8x w/RandAug [35]	88.1	77.3	85.0	82.3
YOLOv8x w/X-Paste [42]	75.3	89.1	84.1	81.6
YOLOv8x w/Copy-Paste [9]	87.4	77.8	87.9	82.3
YOLOv8x w/ARCP	**90.8**	**91.0**	**96.2**	**90.9**

* ↑ indicates that the higher the number, the better the performance. * Bold indicates the best number in this table.

**Table 3:** Accuracy in YOLOv8x [53] on the FieldSAFE background dataset created with ARCP.

Model	Precision↑ (%)	Recall↑ (%)	AP↑ (%)	F1 Score↑ (%)
YOLOv8x [53]	25.3	28.9	13.5	26.6
YOLOv8x w/RandAug [35]	34.0	22.3	13.2	26.9
YOLOv8x w/X-Paste [42]	31.6	28.7	18.6	30.1
YOLOv8x w/Copy-Paste [9]	54.9	66.2	44.6	60.0
YOLOv8x w/ARCP	**82.9**	**63.8**	**72.3**	**72.1**

* ↑ indicates that the higher the number, the better the performance. * Bold indicates the best number in this table.

**Table 4:** AP accuracy in YOLOv7x [58] by training on multiple fallen person detection datasets with the FieldSAFE [60] dataset as background.

Model	(**a**)	(**b**)	(c)	(**b**) + (c)	(**a**) + (c)
YOLOv7x [58]	37.9	32.9	30.7	32.1	38.5
YOLOv7x w/Copy-Paste [9] (**a**)	-	66.2	28.2	64.4	-
YOLOv7x w/Copy-Paste [9] (**b**)	57.1	-	17.6	-	51.3
YOLOv7x w/Copy-Paste [9] (**a**) + (**b**)	-	-	22.9	-	-
YOLOv7x w/ARCP (**a**)	-	**68.2**	**33.1**	**66.2**	-
YOLOv7x w/ARCP (**b**)	**68.7**	-	**36.4**	-	**64.9**
YOLOv7x w/ARCP (**a**) + (**b**)	-	-	**38.0**	-	-

* Bold indicates a value that is increased from the baseline in this table.

**Table 5:** AP accuracy in YOLOv8x [53] by training on multiple fallen person detection datasets with FieldSAFE [60] dataset as background.

Model	(**a**)	(**b**)	(c)	(**b**) + (c)	(**a**) + (c)
YOLOv8x [53]	37.3	37.4	25.4	32.1	35.6
YOLOv8x w/Copy-Paste [9] (**a**)	-	71.9	34.8	70.3	-
YOLOv8x w/Copy-Paste [9] (**b**)	52.2	-	24.4	-	44.6
YOLOv8x w/Copy-Paste [9] (**a**) + (**b**)	-	-	**29.9**	-	-
YOLOv8x w/ARCP (**a**)	-	**78.0**	**50.2**	**75.7**	-
YOLOv8x w/ARCP (**b**)	**66.0**	-	**30.5**	-	**60.8**
YOLOv8x w/ARCP (**a**) + (**b**)	-	-	28.0	-	-

* Bold indicates a value that is increased from the baseline in this table.

**Table 6:** Accuracy in YOLOv7x [58] for test set images Copy-Paste with sunlight to a test set with ARCP applied using FieldSASFE [60] as background images.

Model	Precision↑ (%)	Recall↑ (%)	AP↑ (%)	F1 Score↑ (%)
YOLOv7x [58]	**46.6**	27.1	23.6	34.3
YOLOv7x w/Copy-Paste [9]	21.3	24.8	13.0	22.9
YOLOv7x w/ARCP	40.4	**52.5**	**34.2**	**45.7**

* ↑ indicates that the higher the number, the better the performance. * Bold indicates the best number in this table.

**Table 7:** Accuracy in YOLOv8x [53] for test set images Copy-Paste with sunlight to a test set with ARCP applied using FieldSASFE [60] as background images.

Model	Precision↑ (%)	Recall↑ (%)	AP↑ (%)	F1 Score↑ (%)
YOLOv8x [53]	25.0	24.9	12.8	24.9
YOLOv8x w/Copy-Paste [9]	30.8	27.0	14.7	28.8
YOLOv8x w/ARCP	**37.2**	**34.6**	**19.0**	**35.9**

* ↑ indicates that the higher the number, the better the performance. * Bold indicates the best number in this table.

## Data Availability

Data are contained within the article.
